# Different Conditions for the Modification of Polycaprolactone Films with L-Arginine

**DOI:** 10.3390/ijms21196989

**Published:** 2020-09-23

**Authors:** Yuliya Nashchekina, Alina Chabina, Alexey Nashchekin, Natalia Mikhailova

**Affiliations:** 1 Institute of Cytology of the Russian Academy of Sciences, Center of Cell Technologies, Tikhoretsky Pr. 4, St. Petersburg 194064, Russia; chabina-alina@yandex.ru (A.C.); natmik@mail.ru (N.M.); 2 Ioffe Institute, Laboratory “Materials and Structures of Solid State Electronics”, Polytekhnicheskaya Str., 26, St. Petersburg 194021, Russia; nashchekin@mail.ioffe.ru

**Keywords:** polycaprolactone films, arginine, surface functionalization, mesenchymal stem cells

## Abstract

Poly-ε-caprolactone (PCL) is a biodegradable polymer used in regenerative medicine. Mesenchymal stem cells (MSCs) play an important role in the regeneration of different tissues. The hydrophobicity and neutrality of a PCL surface reduce MSCs’ adhesion and proliferation. In this study, PCL films were treated with arginine to improve surface hydrophilicity. The influences of arginine concentration, temperature, and solvent on PCL surface properties were investigated. PCL films treated with a solution of arginine in isopropyl alcohol were found to have the maximum number of amino groups. The greatest number of cells, 2 h after seeding, adhered to such films. It was shown that amino groups affect the interaction of cells with a modified surface and the hydrolysis reaction after treatment with isopropyl alcohol promotes the formation of adhesive focal contacts. Hence, our results illustrate that functional groups on the PCL surface after arginine solution treatment regulate MSC adhesion and focal contact formation.

## 1. Introduction

Recent advances in tissue engineering have made remarkable breakthroughs in artificial regeneration for tissue repair thanks to the emergence of the successful optimization of artificial materials [1,2]. It has been more and more recognized that cell behavior in vitro depends on the surface properties of materials [3,4]. In recent decades, strategies to prepare artificial polymer materials with suitable surfaces for cell adhesion and proliferation have been extensively reported. Poly(lactic acid) (PLA), poly(glycolic acid) (PGA), and polycaprolactone (PCL) have been extensively used for scaffold formation [5]. PCL has good biocompatibility, biodegradability, mechanical strength, and flexibility [6]. Nevertheless, it has low surface wettability due to its hydrophobicity, and the absence of specific binding sites adversely affects cell attachment and proliferation.

One way to overcome these limitations is surface modification of polymer scaffolds. Unlike bulk modification, where the mechanical strength and elasticity of a material change, surface modification allows changes to occur only on the polymer surface where the cells interact, while the bulk properties of the scaffold remain unchanged. Today, many methods have been proposed for surface modification of polymer scaffolds intended for cell cultivation and transplantation. Surface modification of polymers with proteins (fibronectin, collagen, vitronectin, laminin) as a part of the extracellular matrix (ECM) is a regular approach [7]. Although the ECM proteins demonstrate high attachment efficiency [8,9], the whole-protein modification strategy is limited by complexity in purification, immunogenicity, and fast enzymatic degradation [10]. These proteins include a specific tripeptide sequence RGD (arginine−glycine−aspartic acid) that is very specifically recognized by the cell surface receptor protein integrin. However, surface modification of a tripeptide sequence requires functional groups on polymer scaffold surface. However, PCL, like most polyesters, does not have functional groups. There are several methods for polyester modification in order to improve the surface properties of scaffolds for cell cultivation. Surface modification of PCL scaffolds with small chemical groups is a simple and affordable method for improving the polymer surfaces. It was demonstrated that such small chemical groups as hydroxyl and amino groups effect on adhesion, growth, migration, and differentiation of mesenchymal stem cells (MSCs) [11].

Some authors have used oxygen or NH_2_ plasma for surface modification of PCL and found increased surface hydrophilicity, surface energy, and total amount of oxygen or amino functional groups on the PCL surface [12]. Among these methods, aminolysis is more convenient compared to protein coating and peptide modification, and it is clearer in mechanism than plasma treatment. Also, it has a minimum influence on bulk properties.

Aminolysis involves breaking ester bonds of PCL and forming amide bonds, –NH_2_ and –OH groups. The aminolysis reaction depends on a number of factors such as the properties of diamines, reaction temperature, and solvents. Changing these parameters allows one to control the number of amino groups. Despite the fact the aminolysis reaction has been intensively studied in the last 20 years, the mechanism of the influence of amino groups on cells cultured on modified polymers has not yet been clarified. It has been repeatedly shown that the introduction of amino groups promotes cell adhesion and proliferation. Zhu et al. showed that the modification of PCL with 1,6-hexamethylenediamine increases cell adhesion and proliferation [13]. Earlier, we showed the possibility of PCL film aminolysis by arginine [14]. Arginine is an amino acid that has two amino groups; one of these is able to enter into the aminolysis reaction and form a peptide bond, and the second one remains free.

As already noted, such parameters as temperature and polarity of solvents influence the aminolysis process. We have shown that the amount of arginine bound to the polymer surface increases at elevated temperatures. Other authors have shown that the presence of isopropyl alcohol increases the intensity of the aminolysis reaction. However, it should be noted that, in parallel with the aminolysis reaction, the hydrolysis reaction also takes place. As a result, ester bonds also dissolve, and carboxyl COO and hydroxyl OH groups form. Moreover, heating and isopropyl alcohol accelerate the hydrolysis reaction. Currently, we did not find studies on the effect of these two processes on cell behavior.

In the present work, mesenchymal stromal cells were selected to assess the contribution of aminolysis and hydrolysis reactions to the mechanisms of cell interactions with a modified PCL surface.

MSCs have become widely studied over the past ~30 years for their interesting cell biology, broad-ranging clinical potential, and for their role as a central building block in tissue engineering. MSCs grow readily in a culture dish, and they have intrinsic proliferation and differentiation potentials. MSCs produce an abundance of useful growth factors, cytokines, and extra cell matrix protein.

The fate of MSCs after seeding is locally regulated by the new environment and the surface to which they adhere. The influence of aminolysis and hydrolysis reactions on MSCs’ behavior is still missing in the literature. The functional groups formed in the process of aminolysis and hydrolysis reactions on the surface of PCDs primarily affect the adhesion processes. The adhesion of cells to the polymer surface of scaffolds is one of the important stages in the primary response of cells to external factors immediately after seeding. MSC adhesion regulates the further behavior of cells and their participation in new tissue formation. Therefore, studying the effect of surface properties on cell behavior is not only fundamentally important but also an applied task of modern regenerative medicine.

By this in depth study, we shall clarify the following major concerns: (1) how aminolysis reaction conditions, namely temperature and polarity of solvent, affect the amount of arginine bound to PCL; (2) its influence on the surface properties of PCL films; and (3) how the formed functional groups effect the MSCs’ behavior. Understanding these basic principles is of great importance for better use of aminolysis and hydrolysis for designing advanced biomaterials.

## 2. Results and Discussion

**Figure 1:**
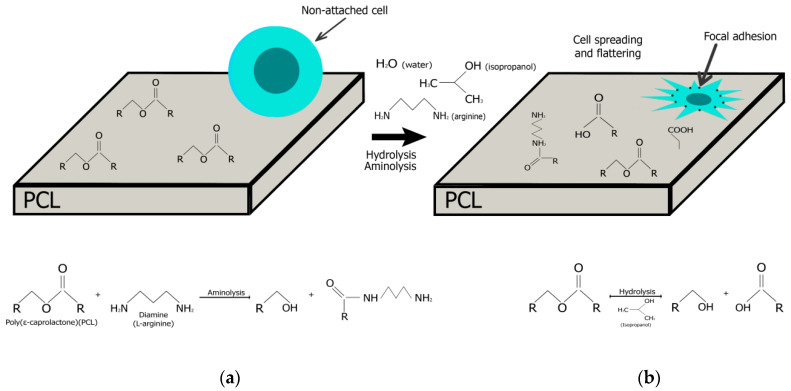
Schematic presentation of chemically based reaction to introduce reactive amine groups on the surface of polymers. Polycaprolactone (PCL) treatment with diamine (L-arginin), resulting in the production primary amines on the material surface (**a**); PCL hydrolysis causing the polymer to break into two components (**b**).

As shown schematically in Figure 1, the creation of active functional groups on the PCL surface depends on a number of factors: as a result of the aminolysis reaction by arginine, free amino groups (-NH_2_), and hydroxyl groups (-OH) are formed on the surface; as a result of the hydrolysis reaction under the influence of isopropanol and water, carboxyl (-OOH) and hydroxyl (-OH) groups are created. The presence of all functional groups increases the hydrophilicity of the surface and hence the attractiveness for cell attachment.

### 2.1. Aminolysis Treatment and Ninhydrin Staining of Free Amino Groups

In principle, aminolysis is a reaction between the amine groups of a diamine and the ester groups of PCL. The aminolysis depends on many factors such as surface morphology of PCL films, polarity of solvents, and reaction temperature.

A large amount of work has been carried out to elucidate the mechanism of aminolysis of model esters [15,16,17]. A positively charged tetrahedral intermediate is formed after nucleophilic attack on the carbonyl carbon of PCL. Under basic conditions, the tetrahedral intermediate is deprotonated, leading to the extremely unfavorable R-NH-leaving group. Under acidic or neutral conditions, the amine leaving group (R-NH2) is strongly favored over the alcohol leaving group (RC-O-), and therefore, the reaction does not carry on. Therefore, aminolysis is generally passes out either in an aprotic polar solvent, such as an alcohol or in basic aqueous solution [18].

Our preliminary results showed that when the films were treated with arginine solution at room temperature, the maximum number of engraft free -NH2 groups was observed after 24 h of film incubation [19]. When PCL films were modified by arginine solution with heating, the maximum number of engraft free –NH2 groups was determined after 1 h of treatment. With a longer exposure to heat treatment, PCL films were significantly degraded. The films are destructed after increasing the treatment time with arginine solution and under heating (40 °C). It is known from the literature that heating at 40 °C PCL is completely hydrolyzed within 1 h [20]. Here, 40 °C is the temperature that the degradation process of the polymer ester bonds actively starts. Free hydroxyl, -OH, and carboxyl, -COOH, groups are formed on the PCL film. After carboxyl group formation the arginine amino groups in the solution begin to interact with the free carboxyl groups.

**Figure 2:**
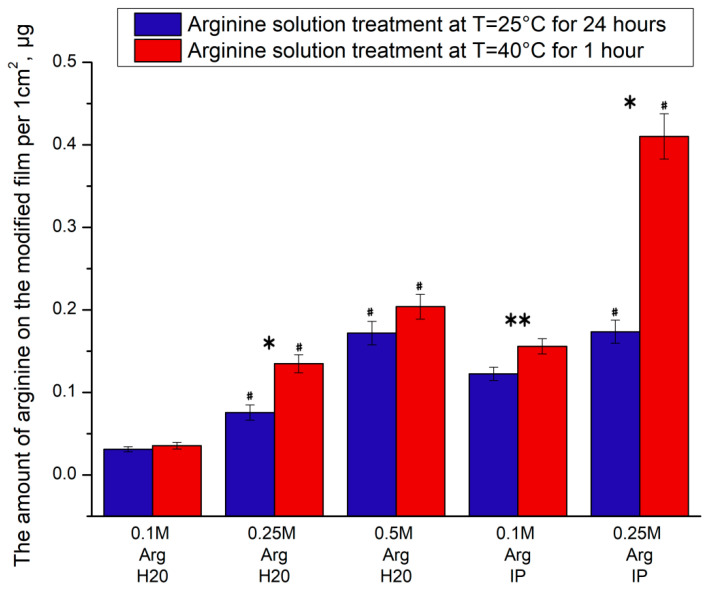
The dependence of the arginine amount on the modified film per 1 cm^2^ on the solution concentration (water (H_2_O) or isopropanol (IP)) at T = 40 °C and T = 25 °C. (*n* = 5: *—*p* < 0.02 for the same concentration data, **—*p* < 0.05; #—*p* < 0.02 compared with lower concentration, but the same temperature).

Processing of films with heating significantly increases the amount of immobilized arginine on the surface of the PCL film (Figure 2). So, when treated with a solution of arginine at a concentration of 0.25 M and heated, the amount of immobilized arginine on a PCL film is three times higher compared to similar samples treated at room temperature. PCL films treated with a solution of arginine at a concentration of 0.5 M and at a temperature of 40 °C have four times more arginine in comparison to similar films processed at room temperature. Films treated with a solution of arginine in the presence of isopropanol also have more amino groups when heated compared to films processed at room temperature. Our results are consistent with the data of Zhu et al., who investigated the PCL aminolysis reaction with a solution of hexamethylenediamine and showed that the maximum number of amino groups are immobilized at a processing temperature of 40 °C [21]. It should also be noted that, in most studies, diamines treated with heat are used to modify PCL scaffolds. The maximum processing temperature does not exceed 30–40 °C [21,22].

The influence of different arginine concentrations and solvent consisted of water and isopropanol (3:1) on the amount of immobilized NH_2_ groups were also investigated. The results demonstrated that the –NH_2_ group density on the PCL films increased along with the growth of arginine concentration in water or in solvent from isopropanol and water (Figure 2).

It has already been repeatedly shown that the number of immobilized amino groups on the surface of PCL films increases with an increase in the concentration of diamine [13,15,19]. However, the concentration of diamine in the solution is limited by the solubility limit of diamine. In our study, arginine is quite soluble in water, but its solubility in isopropanol is limited. Therefore, the modification of PCL films with arginine was carried out in a solution of water and isopropyl alcohol. The ratio of water to alcohol was 3:1. The maximum solubility of arginine in such a solvent does not exceed 0.25 M. The presence of isopropanol in the solvent, as well as heating the reaction solution, allows the maximum amount of amino groups to be immobilized on the surface of the PCL film. At room temperature, the number of immobilized amino groups on films treated in a 0.5 M aqueous solution of arginine is equal to the number of amino groups immobilized on films treated with a 0.25 M solution of arginine in the presence of isopropyl alcohol. However, upon heating, the number of amino groups immobilized on films treated in a 0.25 M solution of arginine in the presence of isopropanol is two times higher compared to films treated in a 0.5 M aqueous solution.

It has already been shown that solvent polarity plays an important role in the aminolysis reaction [19]. The reaction was very slow in water, the most polar solvent in this study due to the poor wetting of the hydrophobic PCL films, which thereby makes it difficult for arginine molecules to approach the PCL molecules. In the relatively less polar alcohols, the reaction rates were much higher, and the highest rate was achieved in isopropanol. It has been previously shown that the rate of aminolysis reaction increases with decreasing solvent polarity, and the maximum number of amino groups is immobilized on the surface of PCL films in the presence of methanol [19]. Indeed, isopropanol is most widely used for aminolysis of PCL scaffolds [21,22].

### 2.2. Fourier Transform Infrared

Fourier transform infrared (FTIR) spectroscopy is a powerful physicochemical technique that allows one to characterize a wide variety of solids, liquids, or gases by measuring their infrared spectra. The main advantages of this non-destructive method are the speed of analysis, as well as high spectral resolution in a wide spectral range. In this paper, FTIR spectroscopy is used to study the modification of polymers by identifying local spectral features corresponding to specific chemical bonds or groups.

**Figure 3:**
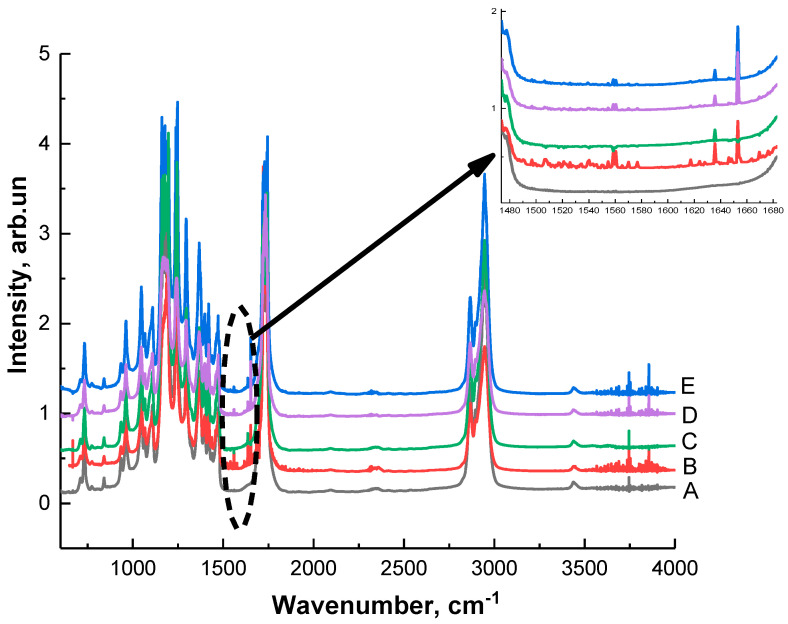
Fourier transform infrared spectra of the various films: (A) unmodified poly(“-caprolactone), (B) PCL treated with the 0.25 M arginine solution in water for 24 h at T = 25 °C, (C) PCL treated with the 0.25 M arginine solution in isopropanol for 24 h at T = 25 °C, (D) PCL treated with the 0.25 M arginine solution in water for 1 h at T = 40 °C, (E) PCL treated with the 0.25 M arginine solution in isopropanol solution for 1 h at T = 40 °C. The two additional peaks in the range from 1550 to 1650 cm^−1^ in aminolyzed PCL films indicated the introduction of amine groups onto the PCL substrates.

Figure 3 shows the Fourier transform infrared (FTIR) spectra of the pristine PCL (A), PCL modified by arginine solutions with different concentrations in water or in solutions from water, and isopropanol at room temperature and with heating. FTIR spectra of PCL films treated with a 0.1 M arginine solution in water at room temperature and heating did not differ from the control; therefore, they are not shown in this figure. For the pure PCL film, we can see the characteristic peaks for carbonyl groups at 1720 cm^−1^ (n(C=O)) and aliphatic groups at 2947 (nas(C–H)) and 2865 cm^−1^ (ns(C–H)). After aminolysis treatment, FTIR spectra of modified PCL films have two additional peaks in the range 1510–1580 cm^−1^, which are assigned to amide II and mainly associated with N–H bending vibrations. The second peaks in the range 1600–1700 cm^−1^ corresponds to amide I, and it is mainly connected with a C=O (carbonyl) stretching vibration (70–85%) and C–N group vibrations (10–20%) [23,24]. The results of FTIR analysis confirm the previously obtained results of the ninhydrin reaction.

### 2.3. SEM Analysis

**Figure 4:**
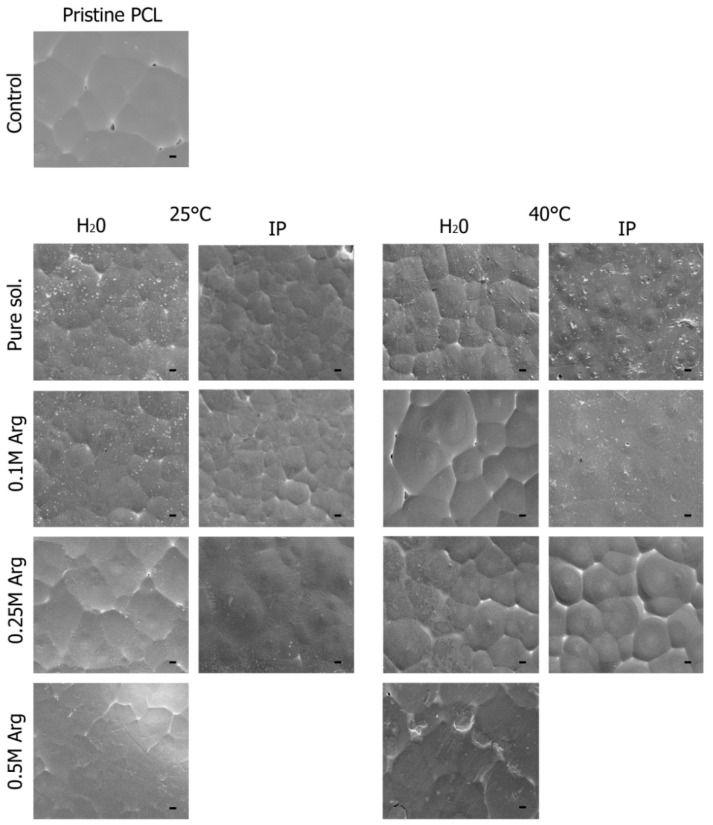
Scanning electron microscopy (SEM) images of PCL films; unmodified PCL film; film treated with 0.1 M arginine solution for 1 h at T = 40 °C; film treated with 0.25 M arginine solution for 1 h at T = 40 °C; film treated with 0.5 M arginine solution for 1 h at T = 40 °C; film treated with 0.1 M arginine solution for 24 h at T = 25 °C; film treated with 0.25 M arginine solution for 24 h at T = 25 °C; film treated with the 0.5 M arginine solution for 24 h at T = 25 °C. Scale bar 100 µm.

The changes in the morphology of the PCL film surfaces after each modification were characterized by scanning electron microscopy (SEM). Figure 4 shows the representative SEM images of the pristine PCL and modified PCL surfaces. The pristine PCL film displayed a relatively uniform and smooth surface including little peaks and troughs. This is typical for PCL membranes which are prepared using the same procedure [25]. No obvious morphology change was found after aminolysis (Figure 4). Despite the effects of heat treatment, as well as the influence of isopropyl alcohol, no noticeable changes in the morphology of modified films were observed in our study or in the works of other authors [20,25].

### 2.4. Interaction between Cells and Substrate

It is widely acknowledged that surface hydrophilicity and surface charge have a significant impact on cell behaviors such as adhesion, migration, proliferation, and extracellular matrix protein synthesis. Webb and Ruardy pioneered the study in this field, and they demonstrated that cells prefer to attach to the surfaces with intermediate hydrophilicity [26,27]. Surfaces with a water contact angle of around 40 are better for cell adhesion. However, surface hydrophilicity is not the most important factor influencing cells’ behavior. The ability of cells to adhere and proliferate on different polymer surfaces could be related to both hydrophilicity and surface chemistry, which have the most influence on cell response [28,29]. Accordingly, it was necessary to find a good compromise between these two factors to enhance the response of the MSCs.

**Figure 5:**
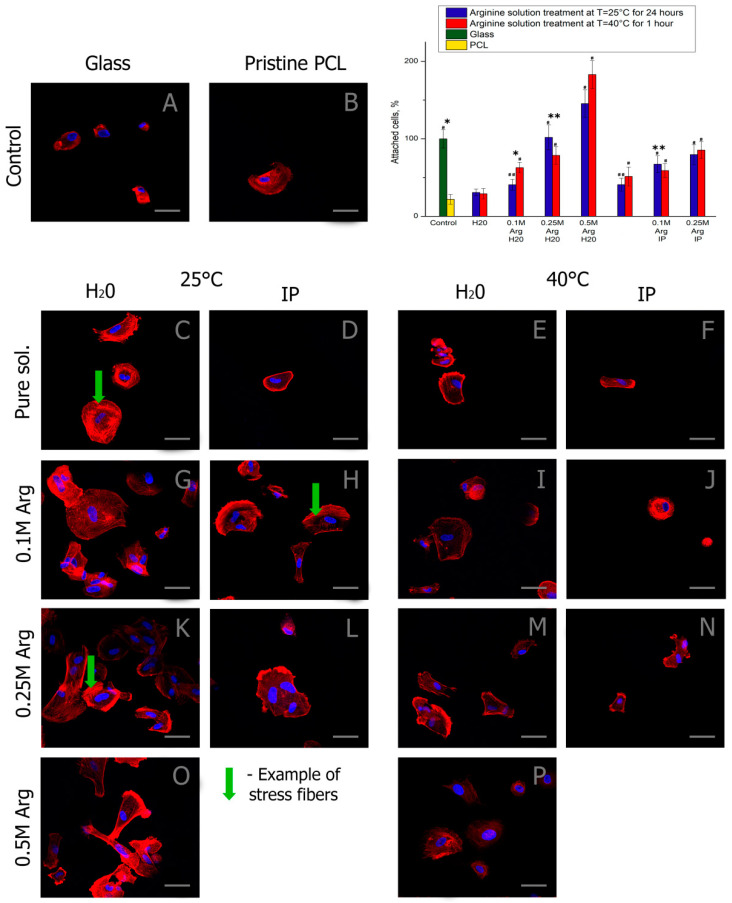
Fluorescence micrographs of Mesenchymal stem cells (MSCs) with actins (red) and nuclei (blue) stained after 2 h cultivation: (**A**) on the pure glass, (**B**) on the pristine PCL, (**C**) on the film treated with water for 24 h at T = 25 °C, (**D**) on the film treated with water/isopropanol (3:1) for 24 h at T = 25 °C, (**E**) on the film treated with water for 1 h at T = 40 °C, (**F**) on the film treated with water/isopropanol (3:1) for 1 h at T = 40 °C, (**G**) on the film treated with the 0.1 M arginine solution in water for 24 h at T = 25 °C, (**H**) on the film treated with the 0.1 M arginine solution in water/isopropanol (3:1) for 24 h at T = 25 °C, (**I**) on the film treated with the 0.1 M arginine solution in water for 1 h at T = 40 °C, (**J**) on the film treated with the 0.1 M arginine solution in water/isopropanol (3:1) for 1 h at T = 40 °C, (**K**) on the film treated with the 0.25 M arginine solution in water for 24 h at T = 25 °C, (**L**) on the film treated with the 0.25 M arginine solution in water/isopropanol (3:1) for 24 h at T = 25 °C, (**M**) on the film treated with the 0.25 M arginine solution in water for 1 h at T = 40 °C, (**N**) on the film treated with the 0.25 M arginine solution in water/isopropanol (3:1) for 1 h at T = 40 °C, (**O**) on the film treated with the 0.5 M arginine solution in water for 24 h at T = 25 °C, (**P**) on the film treated with the 0.5 M arginine solution in water for 1 h at T = 40 °C, Statistics of cell adhesion on surfaces with different functional groups after 2 h cultivation. (*n* = 5: *—*p* < 0.01, **—*p* < 0.05 for the same concentration data, #—*p* < 0.01, ##—*p* < 0.05 compared with the unmodified PCL). Scale bar 50 µm.

It is important to investigate MSCs adhesion during the first hours after seeding. The cytoskeleton organization of MSCs on the pristine and arginine modified PCL films were studied further. As shown in Figure 5, compared to those on the pristine PCL films, the cells cultured on the PCL surfaces modified with arginine in water showed more stressed actin fibers, suggesting better cell adhesion. This is consistent with the results in Figure 5B. The higher the concentration of arginine in the solution, the more cells adhere to the arginine modified PCL surface. The largest number of cells adhered to PCL films modified with a 0.5 M arginine solution in water. Despite the fact that the presence of isopropanol in arginine solution significantly increases the amount of arginine bound to the PCL surface with heating (Figure 2), the number of cells and their spreading on PCL films treated with isopropanol solution are less than on PCL films treated with an arginine solution in water. This may be due to the partial toxic effect of residual isopropanol, which is adsorbed on the surface of the films. Other authors have not provided similar studies on the effect of isopropanol on cell adhesion, despite the fact that the aminolysis reaction by diamines is carried out only in the presence of isopropanol [24,30]. The MSCs on the PCL surfaces modified with arginine in water showed a random organization of cell cytoskeleton without a preferential direction. In our work, as in previously published studies of other authors, the presence of amino groups on the surface of PCL increases the number of adherent cells.

After one day of cultivation, the cells in all samples were well flattened and had the elongated fibroblast-like shape characteristic of MSCs. Thus, a high concentration of arginine on the PCL surface promotes cell adhesion to the modified surface in the first hours after seeding, but it reduces proliferative activity during longer cultivation. The optimal amount of arginine on the film surface is 0.1 μg per 1 cm of surface.

**Figure 6:**
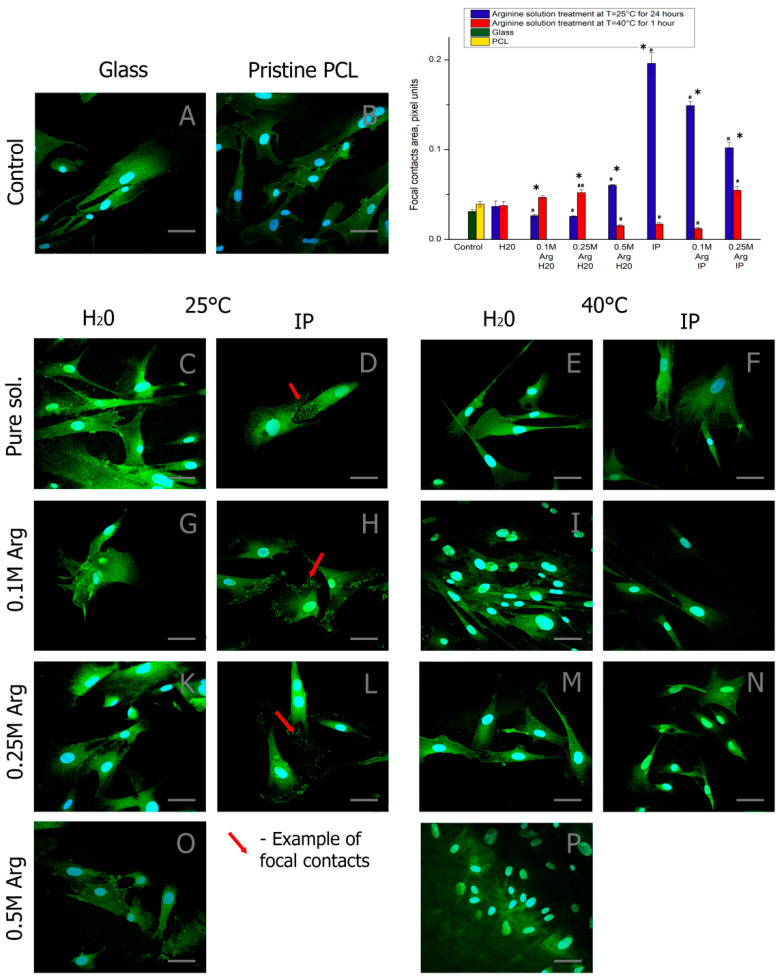
Fluorescence micrographs of MSCs with vinculin (green) and nuclei (blue) stained after three days of cultivation: (**A**) on the pure glass, (**B**) on the pristine PCL, (**C**) on the film treated with water for 24 h at T = 25 °C, (**D**) on the film treated with water/isopropanol (3:1) for 24 h at T = 25 °C, (**E**) on the film treated with water for 1 h at T = 40 °C, (**F**) on the film treated with water/isopropanol (3:1) for 1 h at T = 40 °C, (**G**) on the film treated with the 0.1 M arginine solution in water for 24 h at T = 25 °C, (**H**) on the film treated with the 0.1 M arginine solution in water/isopropanol (3:1) for 24 h at T = 25 °C, (**I**) on the film treated with the 0.1 M arginine solution in water for 1 h at T = 40 °C, (**J**) on the film treated with the 0.1 M arginine solution in water/isopropanol (3:1) for 1 h at T = 40 °C, (**K**) on the film treated with the 0.25 M arginine solution in water for 24 h at T = 25 °C, (**L**) on the film treated with the 0.25 M arginine solution in water/isopropanol (3:1) for 24 h at T = 25 °C, (**M**) on the film treated with the 0.25 M arginine solution in water for 1 h at T = 40 °C, (**N**) on the film treated with the 0.25 M arginine solution in water/isopropanol (3:1) for 1 h at T = 40 °C, (**O**) on the film treated with the 0.5 M arginine solution in water for 24 h at T = 25 °C, (**P**) on the film treated with the 0,5 M arginine solution in water for 1 h at T = 40 °C. Statistics of cell adhesion on surfaces with different functional groups after 1 day cultivation. (*n* = 5: *—*p* < 0.01, for the same concentration data, #—*p* < 0.01, ##—*p* < 0.05 compared with the unmodified PCL). Scale bar 50 µm.

To further examine the expression of vinculin for focal adhesion was detected for MSCs on all of the substrates shown in Figure 6. MSCs show negligible vinculin expression on pristine PCL substrate after three days of cultivation, which indicates cytoskeletal organization is not mediated by clustering of focal adhesions. No significant increase in vinculin-mediated focal adhesion formation was observed on PCL surfaces treated with arginine solutions in water at room temperature or with heating. This is in spite of the presence of a high amount of arginine on the polymer surface. MSCs on PCL films treated with arginine solutions in isopropanol at room temperature showed significantly higher formation of focal adhesion. Focal adhesions were co-localized with actin fibers on all substrates. This indicates that actin organization was mediated by focal adhesion. No significant vinculin expression was observed from MSCs on PCL films treated with arginine solutions and heating. Although quantitatively insignificant (compared to MSCs on PCL surface), the presence of hard segment induced vinculin expression for MSCs on PCL films treated with arginine solutions in water was observed. MSCs on PCL films treated with arginine solutions in isopropanol formed larger and elongated focal adhesions (≥20 μm), which indicate the formation of matured focal adhesions [31]. Thus, we can note that isopropanol significantly affects the number of adhesive contacts formed. However, as was shown earlier, cells on films modified with a solution of arginine in the presence of isopropanol adhere worse than cells seeded on films modified with a solution of arginine in water.

In this work, there is no direct relationship between the number of adhered cells (Figure 5) and the number of focal contacts (Figure 6) on the amount of arginine on the films (Figure 2). We assume that two factors may be the cause. First, there are still no data in the literature on the optimal number of amino groups on the polymer surface for cell adhesion and proliferation. We fully admit that too many amino groups can negatively affect cell adhesion. Second, isopropyl alcohol during the processing of films can remain on the films and adversely affect cultured cells. Moreover, the sorption of isopropyl alcohol will be greater when the solution is heated. Indeed, we see in Figure 6 that, when the films are treated in isopropyl alcohol solution upon heating, the number of focal contacts is insignificant in comparison with the samples treated in an isopropyl alcohol solution at room temperature.

## 3. Materials and Methods

### 3.1. Film Formation

Polymer films were prepared by casting techniques. PCL polymer powder (Mn = 80,000 g/mol; Sigma-Aldrich, St. Louis, MO, USA) was dissolved in chloroform (Reactiv, Saint-Petersburg, Russia). PCL films with a thickness of 5 μm were prepared by casting 0.02 g/mL PCL chloroform solutions in glass Petri dishes. The films were then dried at 25 °C for 48 h. For full solvent evaporation, the films were dried for 24 h at 35 °C. After that, the PCL films were sterilized using ozone treatment in order to study their cellular interactions.

### 3.2. Aminolysis of PCL Films

The films were cut into pieces of 1 cm × 1 cm with subsequent aminolysation in arginine/water or arginine/water/isopropanol solutions with variable concentrations at a given temperature. Solvent consist from water or from water and isopropanol in relation 3:1. After aminolysis, the PCL films were rinsed with copious Milli-Q water for 20 min at room temperature to remove free arginine and dried at 25 °C.

### 3.3. Determination of Amino Groups

The ninhydrin reaction was used to evaluate the amount of arginine bound to the PCL films. In our previous article, we described the method in detail for the quantification of amino acids in dimethyl sulfoxide (DMSO) (Sigma-Aldrich, St. Louis, MO, USA) [14]. According to this method, the quantification amino acids are determined in a homogeneous solution of PCL. To perform the ninhydrin reaction, the modified PCL films were placed into a glass test tube containing 2.5 mL of DMSO and 0.5 mL of 0.2% ninhydrin solution in DMSO and then treated at 80 °C or 100 °C for 15 min to accelerate the reaction between ninhydrin and –NH2 groups. The solution was then cooled for 40 min at room temperature, and the absorbance of the reaction products was recorded using a PE-5400UF spectrophotometer (ECOCHIM, Saint Petersburg, Russia) at a wavelength of 400 nm to calculate the density of amino groups by referring to a calibration curve.

### 3.4. FTIR

The surface molecular structure of modified PCL samples was analyzed using a Fourier transform infrared (FTIR) spectrometer IRPrestige-21 (Shimadzu, Tokyo, Japan), in transmission mode, in the 4000–600 cm^−1^ range, and with spectral resolution 2 cm^−1^.

### 3.5. Scanning Electron Microscopy

The topology of pristine and modified PCL films was evaluated using a JSM-7001F (Jeol, Tokyo, Japan) scanning electron microscope (SEM).

### 3.6. Cultivation of MSCs

MSCs were isolated from the flat pelvis bones of rabbits. A detailed technique for isolating and culturing cells has been described in a previously published article [18]. MSCs were cultivated in α-minimum essential medium (α-MEM; Lonza, St. Louis, MO, USA) supplemented with 10% fetal bovine serum (FBS; HyClone, St. Louis, MO, USA), 100 U/mL penicillin (Sigma-Aldrich, Steinheim, Germany), and 100 mg/mL streptomycin (Sigma-Aldrich, Steinheim, Germany). Cells were cultivated in a CO2-incubator with an atmosphere of 5% CO_2_ content at 37 °C. For our experiments, cells were used following 2–6 passages, and cells were seeded in Petri dishes at a concentration of 1 × 106 cells/cm^2^.

### 3.7. Fluorescence Staining of MSCs

MSCs were fluorescence stained by rhodamine phalloidin and anti-vinculin antibodies in order to study the effects of arginine modification on MSC adhesion and the presence of focal contacts. Pure glass was used as a positive control, while unmodified PCL film was the negative control.

A precise description of the technique for fluorescent staining of cells was described in our previously published work. Briefly, staining was performed as follows. After the cultivation period, the medium was removed, and the adherent cells were washed with PBS, fixed with a 4% formaldehyde solution (Sigma-Aldrich, Saint Louis, MO, USA). Next, a detergent solution was added to the cells. Rhodamine phalloidin (Thermo Fisher Scientific, Carlsbad, CA, USA) was used to stain the actin and DAPI (ab104139; Abcam, Cambridge, MA, USA) was used to stain the nuclei. The cytoskeleton organization was analysed using a confocal microscope Olympus FV3000 (Olympus Corporation, Tokyo, Japan). Focal contacts were study after cell incubation with anti-vinculin antibodies (ab129002; Abcam, Cambridge, MA, USA). Following this incubation, the goat anti-rabbit IgG (H & L chain) antibodies (ab205718; Abcam, Cambridge, MA, USA) and DAPI were added to cells. Then, using a confocal microscope Olympus FV3000 (Olympus Corporation, Tokyo, Japan), we analyzed the cells for the presence of focal contacts.

### 3.8. Cell and Focal Contacts Counts

To study the effects of the modified PCL films on cellular adhesion and number of focal contacts, cells were cultured for 2 h or 1 day. Five different pictures of fields on each sample were used at a wavelength of 365 nm (DAPI) by a fluorescence microscope—Pascal (Carl Zeiss Jena GmbH, Jena, Germany). The ImageJ program was used to count the nuclei in each picture and also to count the number of focal contacts [32].

### 3.9. Statistical Analysis

All experiments were repeated in 3–5 times. An ANOVA and T-test were performed using Microsoft Excel software to analyze the statistically significant differences between samples. Data were considered to be statistically important when *p* < 0.05.

## 4. Conclusions

Cell interaction with PCL surfaces is one of the most important factors affecting cells in tissue engineering and regenerative medicine. In this study, we analyzed the chemical and biological properties of PCL films after treatment. Reactions of arginine solutions in water or in isopropanol at room temperature or with heating (40 °C) were carried out on PCL surfaces. These treatments changed the surface charge of the PCL films without affecting topography. In vitro tests demonstrated an increase of MSCs attachment to PCL films modified with the highest concentration arginine water solution. Arginine concentration, solvent properties, and temperature influenced cell cultivation on modified PCL films. MSCs cultivated on PCL surfaces modified with isopropanol solutions had the greatest amount of focal contacts. The present study had some new insight into the relationship between surface properties after arginine solution treatment and cell adhesion. These results might be helpful for biomaterial design.
